# Associations between negative COVID-19 experiences and symptoms of anxiety and depression: a study based on a representative Canadian national sample

**DOI:** 10.24095/hpcdp.44.2.03

**Published:** 2024-02

**Authors:** Sandy Rao, Gina Dimitropoulos, Jeanne V.A. Williams, Vandad Sharifi, Mina Fahim, Amlish Munir, Andrew G.M. Bulloch, Scott B. Patten

**Affiliations:** 1 Faculty of Social Work, University of Calgary, Calgary, Alberta, Canada; 2 Department of Psychiatry, University of Calgary, Calgary, Alberta, Canada; 3 Mathison Centre for Mental Health Research & Education, University of Calgary, Calgary, Alberta, Canada; 4 Department of Community Health Sciences, University of Calgary, Calgary, Alberta, Canada; 5 Department of Psychiatry, School of Medicine, Tehran University of Medical Sciences, Tehran, Iran; 6 University of Alberta, Edmonton, Alberta, Canada

**Keywords:** COVID-19, anxiety, depression, mental health, loneliness

## Abstract

**Introduction:**

Amid the widespread impact of the COVID-19 pandemic, a notable increase in symptoms of anxiety and depression has become a pressing concern. This study examined the prevalence of anxiety and depression symptoms in Canada from September to December 2020, assessing demographic and socioeconomic influences, as well as the potential role of COVID-19 diagnoses and related negative experiences.

**Methods:**

Data were drawn from the Survey on COVID-19 and Mental Health by Statistics Canada, which used a two-stage sample design to gather responses from 14689 adults across ten provinces and three territorial capitals, excluding less than 2% of the population. Data were collected through self-administered electronic questionnaires or phone interviews. Analytical techniques, such as frequencies, cross-tabulation and logistic regression, were used to assess the prevalence of anxiety and depression symptoms, the demographic characteristics of Canadians with increased anxiety and depression symptoms and the association of these symptoms with COVID-19 diagnoses and negative experiences during the pandemic.

**Results:**

The study found that 14.62% (95% CI: 13.72%–15.51%) of respondents exhibited symptoms of depression, while 12.89% (95% CI: 12.04%–13.74%) reported anxiety symptoms. No clear differences in symptom prevalence were observed between those infected by COVID-19, or those close to someone infected, compared to those without these experiences. However, there were strong associations between traditional risk factors for depressive and anxiety symptoms and negative experiences during the pandemic, such as physical health problems, loneliness and personal relationship challenges in the household.

**Conclusion:**

This study provides insight into the relationship between COVID-19 and Canadians’ mental health, demonstrating an increased prevalence of anxiety and depression symptoms associated with COVID-19-related adversities and common pre-pandemic determinants of these symptoms. The findings suggest that mental health during the pandemic was primarily shaped by traditional determinants of depression and anxiety symptoms and also by negative experiences during the pandemic.

**Highlights**

- This study investigated the effects of negative experiences during the COVID-19 pandemic on mental health in Canada.
- The study found that 15% of Canadians screened positive for symptoms of depression and 13% for symptoms of anxiety from September to December 2020.
- Negative experiences related to COVID-19, such as physical health difficulties, loneliness and challenges in personal relationships, were associated with elevated depression and anxiety symptoms.
- Future research should investigate mental health needs among groups not included in the dataset, such as children, 2SLGBTQI+ communities, First Nations individuals on reserve and individuals who are precariously housed.

## Introduction

On 4 May 2023, the World Health Organization recategorized COVID-19 from a global public health emergency to an established and ongoing health issue.^1^ Despite this shift, the mental health repercussions of the pandemic remain paramount in the Canadian health discourse. Although extant literature recognizes heightened substance use,^2^ increased suicide ideation^3^ and deteriorated self-rated mental health^4^ during the pandemic, crucial research gaps remain. Specifically, the direct and indirect associations between anxiety and depression symptoms and individual COVID-19 experiences, including being diagnosed with COVID-19 oneself or the diagnosis of one’s family members, friends or other close acquaintances, remain understudied.^5^ Moreover, much attention has been focussed on specialized populations, inadvertently overlooking the broader general population.^6^

Despite the overarching stresses attributed to the pandemic, the precise facets of the specific, negative COVID-19 experiences contributing to this stress remain a knowledge gap. By emphasizing symptoms of anxiety and depression over clinical diagnoses, this study adopts an upstream approach, proactively identifying burgeoning mental health challenges that, if unaddressed, may evolve into formal mental illnesses. We sought to advance our understanding of the effects of COVID-19 and associated experiences on the mental health of Canadians. We employed a nationally representative sample to estimate the prevalence of anxiety and depression symptoms and identify associated factors that may have increased the risk of symptoms during this period of heightened stress.

COVID-19 national datasets such as the one we used provide symptom rating scales, serving as validated tools for tracking mental health trends. These datasets allow for the examination of depression and anxiety symptom ratings via self-report screening instruments, thereby monitoring the prevalence of mental health–related symptoms. Although these ratings do not confirm clinical diagnoses, they possess validated cut-points clinicians interpret as signals for further assessment.^7^-^9^ Importantly, even without a formal diagnosis, these symptoms may cause significant distress, compromise well-being and quality of life and thus highlight the potential value of mental health assessment for those with elevated symptoms.

The study had three primary objectives: (1) to estimate the prevalence of anxiety and depression symptoms in the Canadian population between 11 September 2020 and 4 December 2020; (2) to explore the characteristics of this subgroup, including having been diagnosed with or having been in contact with someone diagnosed with COVID-19; and (3) to identify negative COVID-19-related factors associated with positive screens for anxiety and depression symptoms.

## Methods

***Data source***

This study used data from the Survey on COVID-19 and Mental Health (SCMH) conducted by Statistics Canada and made available to researchers through the Canadian Research Data Centre Network.^10^ Therefore, no additional ethics review is required under Article 2.2 of the Tri-Council Policy Statement 2, 2022.^11^ The purpose of the SCMH was to collect data to assess the experiences of COVID-19 on Canadians’ mental health and well-being. Detailed methodological information on the SCMH is available from the Statistics Canada archive.^12^ Briefly, the target demographic of the survey comprised Canadian residents aged 18 years and older, living across all 10 provinces and the territorial capitals of Canada, and excluded less than 2% of the population (those living on-reserve, those in institutions and members of collectives). 

The survey used a two-stage, cross-sectional design, with dwellings as the first stage sampling unit and individuals as the second stage. Stratified by geographic region, a simple random sample of dwellings was selected within each province and three territorial capitals. Data collection occurred between 11 September 2020 and 4 December 2020. Data were collected directly from survey respondents through a self-administered electronic questionnaire or computer-assisted telephone interview. With a response rate of 53.3%, the survey gathered data from 14689 respondents. 

***Measures***

**Demographic variables**

Demographic variables included sex (male, female); age (18–24, 25–44, 45–64, 65+ years); household composition (family and/or others, live alone); income—total annual household (less than CAD 40000, 40000–79999, 80000–99999, 100000–149999, 150000+); education (high school or less, bachelor degree or less, above a bachelor degree); place of residence (urban, rural); employment—during that week, (did not work, did work); province of residence (British Columbia, Alberta, Saskatchewan/Manitoba, Ontario, Quebec, Eastern [New Brunswick, Nova Scotia, Prince Edward Island, Newfoundland and Labrador], Northern [Yukon, Northwest Territories, Nunavut]); and minoritized (yes, no). 

The minoritized variable is a derived variable from the SCMH. Respondents were first self-selected into predefined categories representing various ethnic groups. Thereafter, a second related variable was established in the SCMH as a binary indicator of whether respondents identified themselves as minoritized. We avoided using the terms “visible minority” and “marginalized” because they can perpetuate stereotypes and imply that certain groups are inherently less capable or in need of protection. Instead, we used the terms “minoritized” and “minoritization” to acknowledge that systemic inequalities and oppression place individuals into a “minority” status rather than their characteristics.^13^

COVID-19 occupational variables were also included in this study. Respondents were asked if they were considered a COVID-19 frontline worker or COVID-19 essential worker during the past week (yes, no). 

**Mental health outcomes**

The Patient Health Questionnaire-9 (PHQ-9) and General Anxiety Disorder-7 (GAD-7) were used to assess current (past two weeks) symptoms associated with major depressive disorder (did not meet cut-point, met cut-point) and generalized anxiety disorder (did not meet cut-point, met cut-point). A positive screen on these scales occurs at a cut-point of 10, which would typically justify further assessment in clinical practice.^7^-^9 ^

**Diagnosis with COVID-19**

Respondents to the 2020 SCMH were asked, “Have you or anyone you know been diagnosed with COVID-19?” Response options were yes or no. If the respondent answered yes, the question stem would include, “Who has been diagnosed with COVID-19?” Response options included: yourself (yes, no); another household member (yes, no); a close friend or family member outside of your household (yes, no); a co-worker or colleague (yes, no); someone else with whom you interact with in your community, e.g. neighbour, grocery store worker, babysitter (yes, no); or other (yes, no). Respondents could select multiple categories.

**COVID-19 negative experiences**

Respondents to the 2020 SCMH were asked, “Have you experienced any of the following impacts due to the COVID-19 pandemic?” Response options were: loss of job or income (yes, no); difficulty meeting financial obligations or essential needs, e.g. rent or mortgage payments, utilities, groceries (yes, no); death of a family member, friend or colleague (yes, no); feelings of loneliness or isolation (yes, no); emotional distress, e.g. grief, anger, worry (yes, no); physical health problems, e.g. weight gain or loss, high blood pressure, headaches, sleep problems (yes, no); challenges in personal relationships with members of your household, e.g. children, spouse, parent, grandparents (yes, no). Respondents could select multiple categories.

***Data analysis***

Data analysis was carried out at the Prairie Regional Research Data Centre at the University of Calgary using the statistical software Stata, version 16.0 (StataCorp, College Station, TX, US). To account for the survey design and to provide results that are representative at the national level, estimates were weighted using a set of replicate sampling weights provided by Statistics Canada.^12^ Standard errors, coefficients of variation and 95% confidence intervals were estimated using a master weight and replicate bootstrap weights.^14^ The calculation of replicate bootstrap weights includes adjustments for nonresponse. 

Descriptive techniques were used for the cross-sectional data, including estimating frequencies, to understand the basic distributions of our variables of interest, including the prevalence of anxiety and depression symptoms. Logistic regression models were employed to explore associations between symptoms of anxiety or depression and demographic variables, various COVID-19 diagnosis categories and negative experiences related to COVID-19, such as job loss or the death of a family member. Each COVID-19 diagnosis category and negative COVID-19 experience was considered a separate exposure variable. Statistical significance for the associations was assessed using Wald tests for coefficients from logistic regression analysis using the replicate bootstrap weights. *P* values of less than 0.05 were considered significant.

For COVID-19 diagnosis categories, individuals not diagnosed or who did not know someone diagnosed with COVID-19 constituted the reference group. Unadjusted odds ratios (ORs) and 95% confidence intervals (CIs) were initially estimated. Age- and sex-adjusted logistic regression models were then fit to compare the prevalence of symptoms in COVID-19 diagnosis categories. 

For negative COVID-19 experiences, individuals who did not have those experiences constituted the reference group. Following the derivation of unadjusted ORs, interaction effects of negative experiences with sex and age as determinants of depression and anxiety symptoms were examined. For “physical health problems” and “death of family/friend/colleague,” stratification by age groups (18–44 and 45+ years) was performed due to interactions of these experiences with age. In the absence of age and sex interactions, the COVID-19 experiences exposure variables were adjusted for age and sex. 

Additional statistical modelling was performed to evaluate the effect of covariates on the age- and sex-adjusted odds ratios. A series of logistic regression models were used to generate adjusted ORs and their 95% CIs. These models included covariates identified a priori, including sex, age, household composition, place of residence, employment and minoritization—these covariates were coded as outlined above, except that income, education and province of residence were included after dummy coding, with “CAD 150000 or more,” “greater than bachelor’s degree” and “Quebec” set as the baseline categories. The mode of data collection (self-administered electronic questionnaire, computer-assisted telephone interviewing) was found to have a significant association with symptoms of depression (*p*<0.001). Therefore, it was added to the models with self-administered electronic questionnaire as the referent group. 

Although emotional distress was included in the SCMH as a negative COVID-19 experience, it was not incorporated into the analysis of anxiety or depression symptoms because it is a component of anxiety and depression assessments. There were minimal missing data (less than 10% for any variable), and regression models included only complete cases, forgoing imputation.

## Results

***Prevalence of anxiety and depression symptoms (study objectives 1 and 2)***

The descriptive characteristics of the 14689 Canadians eligible for this analysis are summarized in Table 1. The study found that 12.89% of Canadians screened positive for anxiety symptoms, and 14.62% screened positive for symptoms of depression. The prevalence of anxiety symptoms was found to be elevated in females (15.79%) compared to males (9.86%); in individuals aged 18 to 24 years (20.52%) compared to older age groups (Table 1); in households with family or others living together (13.15%) compared to those living alone (11.40%); in those reporting household income less than CAD 40000 (14.90%) and CAD 40000 to CAD 79999 (14.15%) compared to individuals with a household income of CAD 150000 or more (11.38%); in individuals that were unemployed (14.87%) compared to employed individuals (12.62%); and in frontline workers (17.48%) compared to individuals not working on the front line (11.81%). 

**Table 1:** National prevalence of anxiety and depression symptoms by demographic and socioeconomic variables in the context of COVID-19 (September to December 2020), Canada

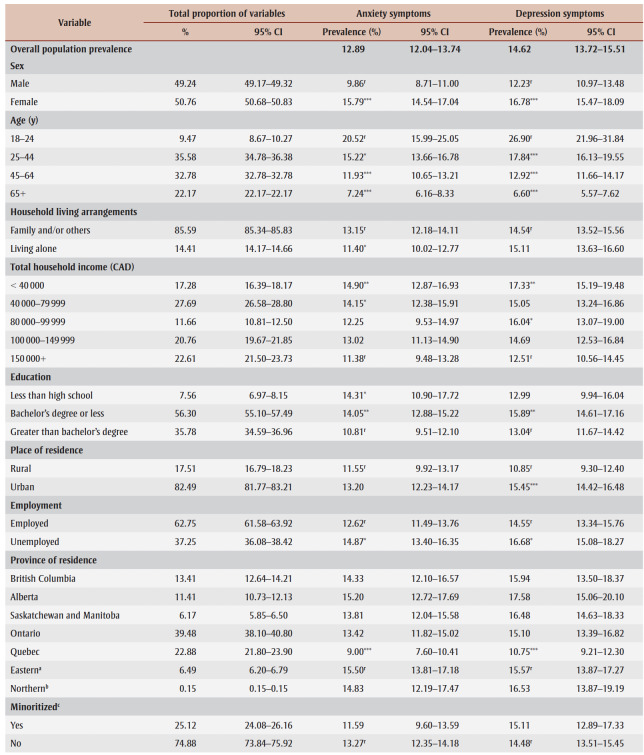 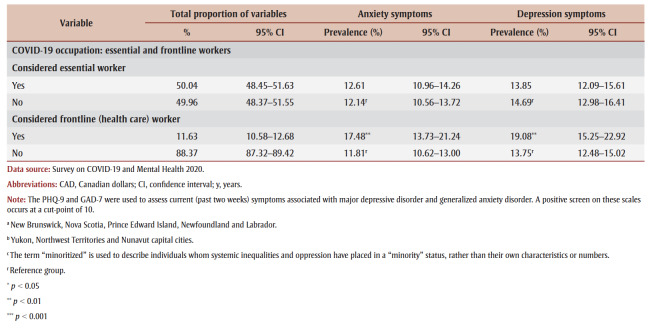

Similarly, the frequency of elevated depression symptoms was highest in females (16.78%) compared to males (12.23%); in individuals aged 18 to 24 (26.90%) compared to older adults (Table 1); in those reporting household income less than CAD40000 (17.33%) and between CAD80000 and CAD99999 (16.04%) compared to individuals with a household income of CAD150000 (12.51%); in individuals with less than a bachelor’s degree (15.89%) compared to individuals with greater than a bachelor’s degree (13.04%); in individuals living in urban centres (15.45%) compared to individuals living in rural areas (10.85%); in unemployed individuals (16.68%) compared to employed individuals (14.55%); and among frontline workers (19.08%) compared to individuals not working on the frontline (13.75%). 

Notably, residents of Quebec had the lowest prevalence of anxiety symptoms (9.00%) and depression symptoms (10.75%) compared to all other Canadian provinces and territorial capitals (Table 1).

***COVID-19 diagnosis categories and associations with anxiety and depression symptoms (study objective 2)***

We examined the association between COVID-19 diagnosis categories (yes, no) and the presence of symptoms of anxiety and depression (met cut-point/did not meet cut-point) using odds ratios as shown in Table 2. There was not a statistically significant association between having received a COVID-19 diagnosis oneself, or any of the subsequent categories, and symptoms of anxiety and depression (*p*>0.05).

**Table 2:** ORs, adjusted ORs, p values and 95% CIs for the associations between COVID-19 diagnosis categories and anxiety and depression symptoms

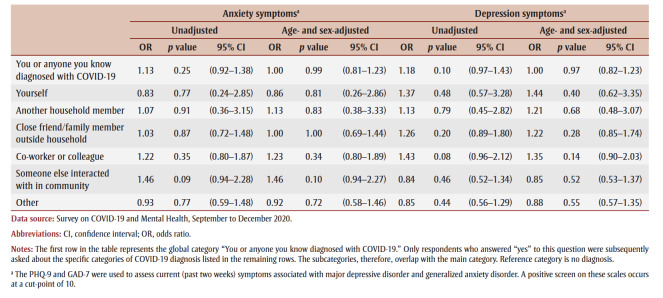

***COVID-19-related factors and association with positive screens for anxiety and depression symptoms (study objective 3)***

Table 3 presents the prevalence and associated CIs of anxiety and depression symptoms corresponding to different negative COVID-19 experiences. Among Canadians that reported negative COVID-19 experiences, the experience associated with the highest prevalence of anxiety (71.08%; 95% CI: 67.84–74.32) and depression (74.14%; 71.19–77.10) symptoms was feelings of loneliness and isolation. Physical health problems related to COVID-19 had the next highest prevalence of anxiety (56.71%; 53.26–60.15) and depression (60.51%; 57.26–63.76) symptoms. The death of a family member, friend or colleague had the least prevalence of anxiety (10.98%; 8.66–13.30) and depression (10.88%; 8.66–13.10) symptoms.

**Table 3:** Frequency of negative COVID-19 experiences in the general population, and with and without symptoms of anxiety and depression

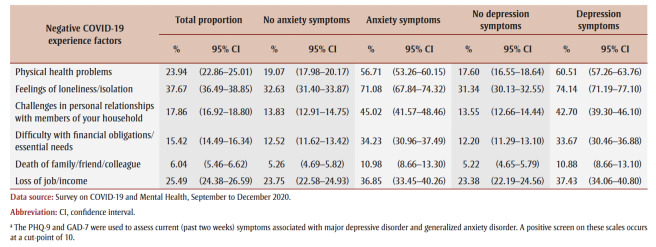

Table 4 presents the adjusted ORs for anxiety and depression symptoms, controlling for all covariates simultaneously. Unadjusted ORs are also included for comparison. The ORs are presented in relation to different negative COVID-19 experiences. In both unadjusted and adjusted analyses, each of the listed COVID-19 experiences demonstrated a statistically significant relationship with symptoms of anxiety and depression (*p*<0.05). For both symptoms of anxiety and depression, physical health problems, loneliness and challenges in personal relationships with members of one’s household had the largest ORs.

**Table 4:** Unadjusted and adjusted ORs for a positive screen of anxiety and depression symptoms, by negative COVID-19 experience factor, with 95% CIs

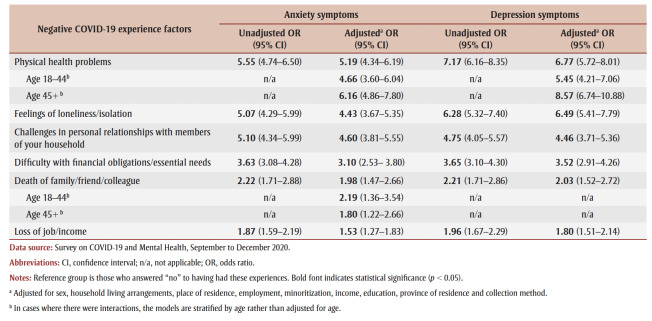

## Discussion

In this study, we investigated the prevalence of anxiety and depression symptoms amid the COVID-19 pandemic, focussing on the specific characteristics of Canadians with elevated symptoms, including COVID-19 diagnosis and COVID-19-related adverse experiences, from September to December 2020. Our findings reinforce that there is a substantial mental health burden associated with the pandemic,^4^,^15^,^16^ underlining the necessity to formulate comprehensive and strategic mental health interventions.

The results highlight variations in the prevalence of anxiety and depression symptoms across gender, age and socioeconomic strata, consistent with other pandemic-related studies.^17^,^18^ Notably, an elevated prevalence of symptoms was detected among women and younger cohorts (aged 18–24 years). The literature suggests an increased gender-based susceptibility to adverse psychological outcomes, observable during the pandemic’s periods of both elevated stress and recovery.^18^,^19^ This may be due to a convergence of stressors such as traditional gender roles, parental responsibilities, labour disparities and a societal environment conducive to violence against women. The shift to home-schooling and the need to care for older individuals exacerbated the burden of care, borne predominantly by women.^20^ Coupled with reduced support systems, these stressors likely worsened poor mental health outcomes in women. Additionally, the tendency of women to report symptoms more than men underscores the need for additional research to identify and address the factors contributing to these gender disparities.

The literature supports the notion that there has been an outsized impact of mental health challenges on young adults during the pandemic.^4^,^21^ Complications unique to this group include educational and employment disruptions,^22^ as is also highlighted in research focussed on youth.^23^,^24^ However, how pandemic-related stressors exacerbate normative stressors associated with academic and professional transitions remains unclear. Nevertheless, a subset of young adults reported enhanced well-being in the initial phase of the pandemic, indicating an appreciation for decelerated lifestyles, increased leisure time for hobbies and personal growth and temporary relief from academic and climate change concerns.^25^

Despite these positives, emerging adulthood remains a period of vulnerability for the onset of mental health disorders. In the pandemic context, academic disruptions, economic hardships, social isolation, misinformation on social media and restricted access to physical activities have likely intensified the mental health challenges faced by young adults.^24^,^25^

Individuals with lower income or who were unemployed reported a higher prevalence of anxiety and depression symptoms, potentially linked to financial insecurities related to the pandemic. This is in contrast to some research that suggests interventions such as financial support and rent bans may have mitigated some mental health impacts.^17^ Geographical variations in symptom prevalence were also noted; Quebec experienced the fewest anxiety and depression symptoms, indicating potential regional differences in stressors or mental health resources availability.^26^,^27^ Despite there being no significant difference in symptom prevalence between minoritized and nonminoritized groups, the influence of systemic disparities on mental health outcomes cannot be discounted.^28^

Our study highlighted occupational disparities related to the pandemic, revealing a higher prevalence of depressive and anxiety symptoms among frontline workers. However, counterintuitively, health care workers who directly engaged with COVID-19 patients reported lower stress levels than those in non-COVID-19 units.^29^ This may suggest that the uncertainty surrounding the pandemic induced greater stress than direct disease exposure, the latter possibly fostering better coping strategies through increased awareness. Increased anxiety symptoms were also noted among individuals in contact with people diagnosed with COVID-19. Theories of crisis, personal construct and adult models of anxiety provide a lens to interpret these findings, pointing to a perceived threat response triggered by the lack of control and predictability in the pandemic’s early stage.^30^-^32^

Moreover, we found that respondents with physical health problems were significantly more likely to report depressive and anxiety symptoms. This is in line with the disruptions in health care delivery during the pandemic, potentially exacerbating pre-existing chronic conditions. 

We further observed intriguing trends, whereby loneliness was strongly associated with symptoms of depression and anxiety, but solitary living was not (for anxiety symptoms). The data underscore the importance of distinguishing between objective social isolation and subjective feelings of loneliness when formulating interventions to improve mental health outcomes. Programs aimed at mitigating feelings of loneliness through social skills training and fostering meaningful connections may offer more benefit than simply increasing the number of social interactions during crisis periods.^33^,^34^

***Strengths and limitations***

This study utilized a nationally representative dataset, capturing a broad demographic range across Canada. This approach allowed for an exhaustive examination of the relationship between COVID-19-related experiences and the national prevalence of anxiety and depression symptoms. Overcoming the limitations of prior research, which was encumbered by varied sampling frames, inconsistent data collection methods and a reliance on public opinion data, this study provides insights into patterns of prevalence of anxiety and depression symptoms during the COVID-19 pandemic.^35^

Despite these strengths, it is essential to acknowledge inherent limitations. For instance, the cross-sectional survey design restricted our ability to formulate definitive conclusions about the pandemic’s mental health impacts, and cannot support causal inference. Measurement errors may have arisen due to the study’s reliance on self-reported data and retrospective recall. 

Although the study’s framework allows for the identification of associations, it falls short in drawing causal inferences. Therefore, we suggest that future longitudinal investigations be pursued to provide more nuanced insights into the temporal patterns and causal relationships between experiences during public health emergencies and mental health outcomes. Additionally, the study excludes subpopulations, including those experiencing homelessness, residents of First Nations reserves and individuals residing in institutions, that may endorse the highest prevalence of anxiety and depression symptoms. 

The study’s design also did not account for the potential influence of multiple exposures to COVID-19 diagnoses, or the severity of the disease experienced by diagnosed individuals. It overlooked the effect of overlapping personal diagnosis experiences and knowledge of others’ diagnoses. These factors could alter the psychological response to the pandemic, limiting an accurate assessment of its mental health impacts. The study did not incorporate respondents’ pre-existing mental health status, a key component in understanding their mental health responses to the pandemic. And, despite both being linked to negative COVID-19 experiences, the concepts of loneliness and social isolation—commonly understood as the subjective feeling of being alone and the objective state of an individual’s social environment, respectively—are not equivalent; however, they were grouped in the SCMH data, which may complicate interpretation. 

Finally, it must be noted that the findings of this study are specific to the general population during the early stages of the pandemic and do not include subsequent significant developments, such as the emergence of new variants and the introduction of vaccines. This context must be considered when considering the applicability and implications of the study’s findings.

## Conclusion

The aim of this study was to explore the prevalence and characteristics of reported anxiety and depression symptoms among Canadians from September to December 2020, focussing on those with COVID-19 diagnoses and COVID-19-related adversities. Using the SCMH, the study suggests important mental health implications arising from the pandemic and points to disparities across various demographic subgroups.

The findings suggest a potential mental health burden from the pandemic, with signs of increased vulnerabilities indicated among women, younger individuals (aged 18–24 years) and lower-income groups. Regional differences may also suggest local stressors or potential gaps in mental health resources. While a direct association between COVID-19 diagnosis and heightened anxiety or depression symptoms was not clearly established, the data do suggest a strong relationship with negative COVID-19 experiences, highlighting the need for more comprehensive mental health approaches. Variations across age groups, professional sectors and diverse communities offer insight into the heterogeneous influence of the pandemic. 

Though resilience is often observed following disasters, with many individuals avoiding psychopathology and some even discovering new strengths, this study suggests the need for a more nuanced and targeted approach to mental health that extends beyond the immediate physical health impacts of the pandemic. It emphasizes the importance of continued research and monitoring to better understand the enduring mental health implications of the COVID-19 pandemic as a persisting health concern. 

## Acknowledgements

The analysis was conducted at the Prairie Regional Research Data Centre, part of the Canadian Research Data Centre Network (CRDCN).

The services and activities provided by the CRDCN are made possible by the financial or in-kind support of the Government of Canada’s Social Sciences and Humanities Research Council, the Canadian Institutes of Health Research, the Canada Foundation for Innovation, Statistics Canada and participating universities, whose support is gratefully acknowledged.

## Conflicts of interest

The authors have no conflicts of interest to declare.

## Authors’ contributions and statement

AM, SR, JVAW, SBP—conceptualization.

AM, JVAW, AGMB, SBP, SR—methodology.

SR, JVAW, SBP—data curation.

GD, SBP—supervision.

SR, GD, JVAW, VS, MF, AM, AGMB, SBP—formal analysis.

SR, VS, MF, GD, AGMB, SBP—verification.

SR, SBP, GD—writing—original draft.

SR, GD, JVAW, VS, MF, AM, AGMB, SBP—writing—review and editing. 

The content and views expressed in this article are those of the authors and do not necessarily reflect those of the Government of Canada, the CRDCN or its partners.
